# Effect of *NR1D1* on the proliferation and differentiation of yak skeletal muscle satellite cells

**DOI:** 10.3389/fvets.2024.1428117

**Published:** 2024-11-04

**Authors:** Yuqi Zhe, Zhijuan Wu, Sibinuer Yasenjian, Jincheng Zhong, Hui Jiang, Ming Zhang, Zhixin Chai, Jinwei Xin

**Affiliations:** ^1^Key Laboratory of Qinghai-Tibetan Plateau Animal Genetic Resource Reservation and Utilization, Sichuan Province and Ministry of Education, Southwest Minzu University, Chengdu, China; ^2^Sichuan Qinghai Tibet Plateau Herbivore Livestock Engineering Technology Center, Chengdu, China; ^3^State Key Laboratory of Hulless Barley and Yak Germplasm Resources and Genetic Improvement, Institute of Animal Science and Veterinary Research, Tibet Academy of Agricultural and Animal Husbandry Sciences, Lhasa, China

**Keywords:** *NR1D1*, yak, skeletal muscle satellite cells, proliferation, differentiation

## Abstract

The severe conditions at high altitudes, where yaks inhabit, contribute to delayed muscular growth and compromised tenderness of their muscle tissue. Myosatellite cells are responsible for the growth and regeneration of skeletal muscle after birth and have the potential to proliferate and differentiate, its development is closely related to meat quality, and the nuclear receptor gene *NR1D1* is involved in muscle formation and skeletal muscle regulation. Therefore, in order to understand the effect of *NR1D1* on muscle satellite cells, we identified the mRNA expression levels of marker genes specifically expressed in muscle satellite cells at different stages to determine the type of cells isolated. Eventually, we successfully constructed a primary cell line of yak muscle satellite cells. Then we constructed *NR1D1* overexpression vector and interference RNA, and introduced them into isolated yak skeletal muscle satellite cells. We performed qPCR, CCK8, and fluorescence-specific to detect the expression of genes or abundance of proteins as markers of cell proliferation and differentiation. Compared with those in the control group, the expression levels of proliferation marker genes *KI-67*, *CYCLIND1*, and *CYCLINA* were significantly inhibited after *NR1D1* overexpression, which was also supported by the CCK-8 test, whereas differentiation marker genes *MYOD*, *MYOG*, and *MYF5* were significantly inhibited. Fluorescence-specific staining showed that KI-67 protein abundance and the number of microfilaments both decreased, while the opposite trend was observed after *NR1D1* interference. In conclusion, we confirmed that *NR1D1* inhibited the proliferation and differentiation of yak skeletal muscle satellite cells, which provides a theoretical basis for further research on the effect of *NR1D1* on improving meat quality traits and meat production performance of yaks.

## 1 Introduction

Yaks (*Bos grunniens*) are an important livestock on the Tibetan Plateau, providing meat and other necessities for Tibetans. Skeletal muscle is the most abundant muscle tissue among adult animals, is an important source of protein, and is important in maintaining homeostasis, movement, and respiration (1). Skeletal muscle is also the most economically valuable tissue of meat-producing animals, and its yield and quality directly determine the productivity benefits of animals. Therefore, it is important to understand the underlying mechanisms that influence skeletal muscle to enhance yak resistance to harsh conditions and improve yak meat yield and quality.

Skeletal muscle accounts for 75–90% of muscle volume and is composed mainly of Muscle satellite cells (SCs), fibers, immune cells, basement membrane, and nerves (2), all of which are key to maintaining normal physiological muscle function. Muscle development depends on muscle cell formation and adipogenesis (3), and SCs are skeletal muscle progenitors. When activated by myoblasts, SCs multiply and differentiate into new muscle fibers (4). Thus, the development and regeneration of skeletal muscle depends on the maintenance of myoblastic properties of SCs, such as their ability to proliferate, differentiate, and self-renew. The growth and development of skeletal muscle depends on the regulation of many genes and pathways. Artificially changing the regulatory factors can promote muscle development and improve meat quality, and help to achieve the goal of modern animal husbandry. Regulatory factors involved in the activation, proliferation, and differentiation of SCs and their mechanisms have been studied extensively, but the individual interaction mechanisms and signaling pathways among the related factors need further study (5). Recent reports have outlined the regulatory impacts of diverse genes on stem cells (SCs) (6–9). However, there remains a scarcity of studies pertaining to yak SCs.

Nuclear receptor subfamily 1, group D member 1 (*NR1D1*), also known as *REV-ERBα*, is a clock gene in the biological clock system. *NR1D1* was first isolated from rat pituitary tumor cells by Lazar et al. (10). As a transcriptional suppressor, *NR1D1* can inhibit downstream genes. *NR1D1* is distributed in all tissues and organs of the body, and its expression has a circadian rhythm (11–13). Studies have shown that *NR1D1* regulates body metabolism (14, 15), inflammation (16) and cell proliferation (17). It is also highly expressed in skeletal muscle and participates in the regulation of multiple physiological processes through various pathways, including mitochondrial biogenesis, oxidative capacity, myogenesis, and myofiber size in skeletal muscle (18, 19). The absence of *NR1D1* in skeletal muscle can result in increased expression of atrophy genes that are associated with decreased muscle mass and reduced fiber size (18). The lack of *NR1D1* in mice impairs sarcoplasmic reticulum/endoplasmic reticulum calcium ATPase-dependent sarcoplasmic reticulum calcium uptake in skeletal muscle (20), while the overexpression of *NR1D1* can improve mitochondrial respiration and exercise in skeletal muscle (21). These findings collectively demonstrate the crucial regulatory role of *NR1D1* in skeletal muscle function.

This study aims to investigate the role of the *NR1D1* gene in yak tissues and SCs. By cloning and constructing *NR1D1* overexpression vectors and interference RNA, these were transfected into yak SCs. Using quantitative reverse-transcription PCR (qPCR), CCK-8, and specific fluorescent staining, we aim to establish a comprehensive and referenceable system for the isolation and culture of yak skeletal muscle satellite cells. Meanwhile, we aim to elucidate the effects of *NR1D1* on different yak tissues and SCs, while exploring its potential mechanisms for influencing skeletal muscle.

## 2 Materials and methods

### 2.1 Animal materials

The animals used for qPCR tissue experiments were Jiulong yaks sourced from Jiulong County, Ganzi Tibetan Autonomous Prefecture, Sichuan Province. Hearts, livers, spleens, lungs, kidneys, longissimus dorsi muscles, and pectoral muscles were collected from three female yaks. After being rinsed with DEPC water, the tissues were wrapped in aluminum foil, labeled, and quickly immersed in liquid nitrogen for subsequent experimental use. All animal experimental procedures were approved by the Animal Care and Use Committee of Southwest Minzu University and conducted in accordance with relevant guidelines and regulations set by the local animal ethics committee.

### 2.2 Isolation, culture, and induced differentiation of yak SCs

**Figure 1:**
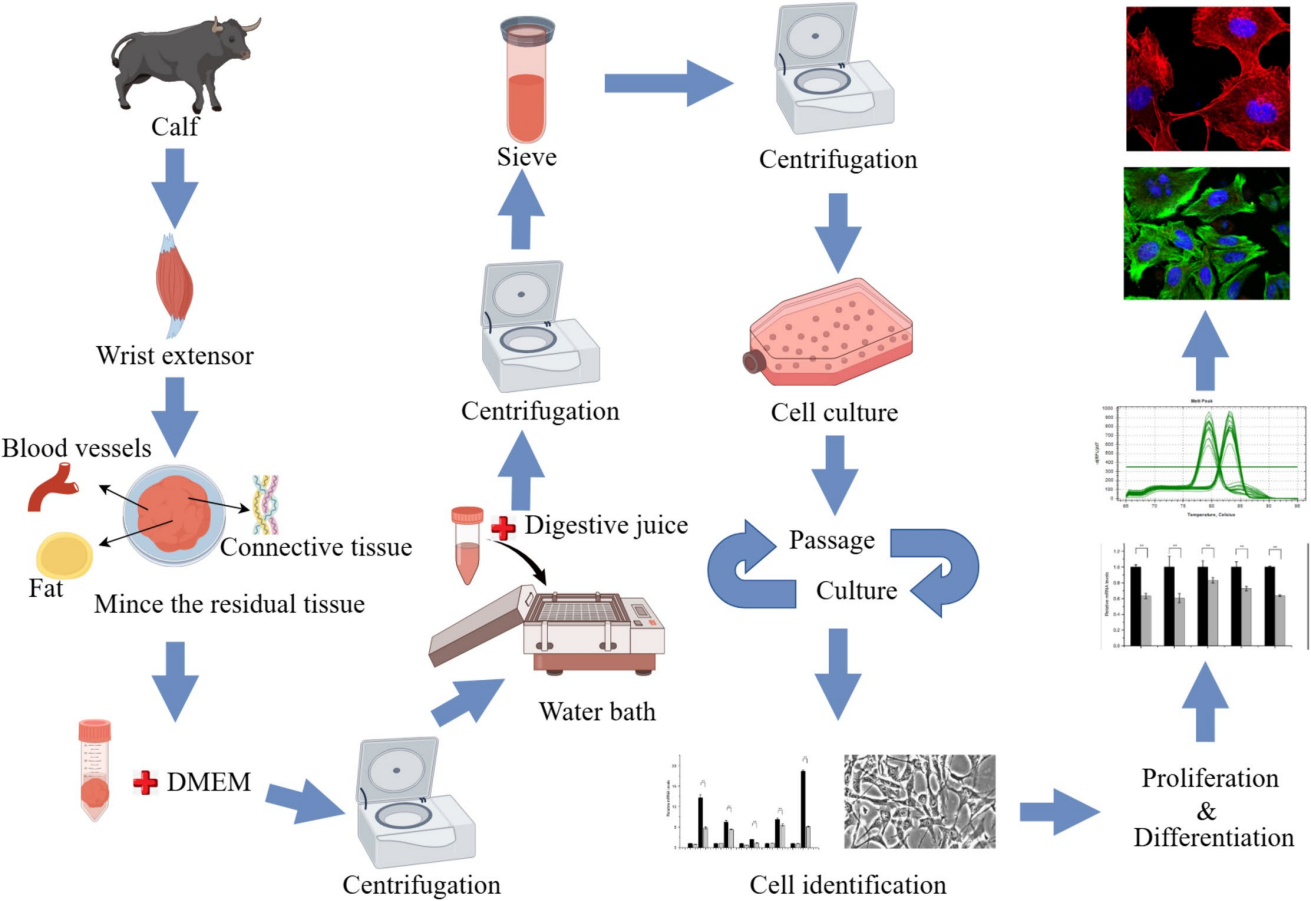
Experimental flowchart (by Figdraw).

The animals used for cell culture were sourced from the Qinghai-Tibet Base Yak Breeding Center of Southwest Minzu University, located in Hongyuan County, Aba Tibetan and Qiang Autonomous Prefecture, Sichuan Province. Two-month-old newborn Maiwa female yak was selected, slaughtered, and samples were collected from their anterior limb skeletal muscle tissue. After partially shaving and sterilizing the yak in the dissection room, the radial carpal extensors were successfully isolated from the animal (Supplementary Figure S1). The sample was immersed in 75% alcohol for 10–15 min and washed 3 times with phosphate-buffered saline (PBS) containing antibiotics, then placed in PBS containing antibiotics, transported on ice, and quickly transferred to the laboratory. In the laboratory, the samples underwent an additional washing process three times with PBS containing antibiotics. The muscle tissue was placed in a petri dish, a small amount of PBS containing antibiotics was added, and the blood vessels, fat and connective tissue were carefully removed. An equal volume of Dulbecco’s Modified Eagle’s Medium (DMEM; Gibco, Waltham, MA, United States), containing antibiotics was added, and subsequently, the sample was minced into shreds. The tissue block was transferred to a 50 mL centrifuge tube with sterilized tweezers or a spoon and centrifuge at 400 × g for 5 min. Add 3 times the volume of digestive solution A precipitated in the previous step (1 mg/mL pronase E, formulated with DMEM containing antibiotics) was added. Digestion was performed at 37°C for 1 h, then the tube was centrifuged at 400 × g for 5 min. The supernatant was discarded and the cell mass was resuspended in the digestive termination solution and left to rest. The supernatant containing SCs was retained. This process was repeated 3 times. The collected supernatant was filtered with a 40-μm cell screen, then centrifuged at 1,000 × g for 10 min to collect cells. The cell clusters were cultured aseptically in a Thermo Scientific incubator (Thermo Fisher Scientific, Waltham, MA, United States) at 37°C and 5% CO_2_. Routine cultures were performed in DMEM with 20% fetal bovine serum (FBS; Gibco), 100 U/mL penicillin-streptomycin, 0.25 μg/mL amphotericin B, and 4 mM glutamine. Adherent cells (mainly myofibroblasts) were cultured for 1 h, and unadherent cells (mainly SCs) were transferred to a new culture bottle for culture. The adherent cells were supplemented with 5 mL–10 mL growth medium. Cells differentiation were induced using induction differentiation medium (DMEM + 2% horse serum (HS; Gibco) + 100 U/mL penicillin-streptomycin + 0.25 μg/mL amphotericin B + 4 mM glutamine) (Figure 1).

### 2.3 Subculture and induced differentiation

After three days of culture in growth medium, the SCs adherent to the culture dish. When the cells were cultured to 70–80%, digested with trypsin, and passaged. On the 6th day of culture, the culture medium was replaced with differentiation medium (2% HS) to induce cell differentiation, which was observed. The mRNA expression levels of *PAX7*, *MYF5*, *MYOD*, *MRF4*, *MEF2C*, *MYMK*, *MYOG*, and *DES* were obtained by qPCR. All primers were synthesized by Tsingke Biotech (Beijing, China) (Supplementary Table S1).

### 2.4 Cloning of *NR1D1*

Primer Premier 5.0 software was employed to design the *NR1D1* primers (Supplementary Table S1), utilizing the *NR1D1* sequence (NM_001078100.2) from GenBank as the template for the design process. The coding sequence (CDS) of *NR1D1* was amplified using yak longissimus dorsi cDNA as the template. PCR products were identified through agarose gel electrophoresis, followed by gel recovery. The recovered products were then ligated into the vector, adhering to the instructions outlined in the pMD^™^ 19-T Vector Cloning Kit (Takara, Otsu, Shiga, Japan). Subsequently, the recombinant plasmids were transformed into *Escherichia coli* DH5α (Accurate Biology, Changsha, China) receptor cells. These cells were incubated overnight on Luria-Bertani (LB) solid medium to allow for colony formation. Positive clones were screened and sequenced for further analysis.

### 2.5 Construction of eukaryotic expression vector

The target fragment was connected to a linearized pcDNA3.1(+) vector using an OKClon DNA linking kit (Accurate Biology), strictly adhering to the manufacturer’s instructions. Subsequently, the recombinant products were transformed and sequenced as previously described. Once the sequencing was confirmed to be accurate, the recombinant plasmid was extracted using an endotoxin-free plasmid extraction kit (Tiangen, Beijing, China) and designated as pcDNA3.1-*NR1D1*.

### 2.6 Cell culture and transfection

SCs were seeded into T25 culture dishes for expansion. After expansion, the cells were digested with trypsin and passaged into 6-well plates at a density of 3 × 10^5^ cells per well. After the cells grew to 70–80% confluence, they were transfected with *NR1D1* overexpression vectors or small interfering RNA (siRNA) and the corresponding control using Lipofectamine 3000 (Invitrogen) according to the manufacturer’s instructions (three wells were transfected in both the experimental group and the control group). After culturing in the proliferation medium for 48 h, the cells were collected, and subsequently, RNA was extracted and reverse transcribed. To detect the mRNA expression levels of *NR1D1*, *NR1D1* siRNA, *KI-67*, *CYCLIND1*, and *CYCLINA*, qPCR was performed. Alternatively, after transfection and when the cell density reached 80–90% confluence, the cells were switched to differentiation medium for 72 h. Following this, the cells were collected, RNA was extracted, and reverse transcription was performed. Subsequently, qPCR was used to detect the mRNA expression levels of *MYOD*, *MYOG*, and *MYF5*.

### 2.7 RNA extraction and quantitative reverse-transcription PCR

Total RNA was extracted from different tissues (approximately 30 mg) or SCs of yaks using TRIzol^®^ reagent (Invitrogen) according to the manufacturer’s instructions. The concentration and quality of the extracted RNA were detected by spectrophotometer (NanoDrop One, United States) and agar gel electrophoresis. Reverse transcription and qPCR of the extracted RNA were performed using a PrimeScript^™^ RT Reagent Kit with gDNA Eraser (Takara) and TB Green^®^ Premix Ex Taq^™^ II (Takara), respectively. To calculate the relative expression levels of mRNA, GraphPad prism 8.0 (GraphPad Software, La Jolla, CA, United States) was used to plot and analyze the significance of the results.

### 2.8 Specific fluorescence staining

Cells were seeded into 24-well plates at a density of 5 × 10^4^ cells per well. When the cells reached 70–80% confluence, the *NR1D1* overexpression vector or *NR1D1* siRNA along with their respective controls were transfected into the cells using lipofectamine 3,000. After transfection, the cells were cultured in proliferation medium for 48 h, or until they reached 80–90% confluence, at which point the medium was changed to differentiation medium for an additional 72 h of culture, the cells were fixed with 4% paraformaldehyde (BL539A, Biosharp) for a duration of 20 min, followed by three washes with phosphate buffered saline containing Tween-20 (PBST), each lasting 5 min. The membrane was broken with 0.5% Triton X-100, then washed twice with PBST for 5 min each time. The cells were sealed with 3% bovine serum albumin (BSA, Invitrogen) at room temperature for 2 h, then incubated overnight with Ki-67 antibody (PA5-19462, Invitrogen) at 4°C. Then, the cells were washed 3 times with PBST for 15 min each time, and the secondary antibody was added at room temperature and the cells were shielded from light for 2 h. The cells were washed 3 times with PBST for 15 min each time or stained with a microfilament red fluorescent probe (C2203S, Beyotime), then incubated with DAPI (Invitrogen) solution away from light for 5 min, and washed twice with PBST for 5 min each time. An inverted fluorescence microscope (Carl Zeiss, Oberkochen, Germany) was used to observe and photograph the cells.

### 2.9 Cell proliferation assay

Cells were seeded into 96-well plates at a density of 3 × 10^3^ cells per well and transfected with the *NR1D1* overexpression vector, *NR1D1* siRNA, or their respective controls. For the Cell Counting Kit-8 (CCK-8, Biosharp) assay, 10 μL of CCK-8 was added to each well at 0 h, 12 h, 24 h, and 48 h post-transfection, followed by incubation at 37°C with 5% CO_2_ for 2 h. Finally, the absorbance values at 450 nm were measured using a microplate reader (Thermo Fisher Scientific).

### 2.10 Statistical analysis

The GraphPad prism 8.0 software (La Jolla, CA, United States) was used to calculate the relative expression level using the 2^−ΔΔCT^ method, and a one-way analysis of variance and multiple comparison test were performed to determine the statistical significance of each group (*p* < 0.05) and visualize the data. For KI-67, DAPI-specific fluorescent staining cells and microfilaments, the average fluorescence intensity was statistically analyzed using ImageJ software for quantification, and the results were visualized using GraphPad prism 8.0 software.

## 3 Results

### 3.1 *NR1D1* is highly expressed in muscle tissue

*NR1D1* is distributed in various tissues and organs of many organisms and has many functions. To determine the expression the expression of *NR1D1* in yak tissues. To determine the expression of *NR1D1* in yak tissues, RNA was extracted from heart, liver, spleen, lung, kidney, longissimus dorsi muscle, and pectoralis muscle and converted into cDNA as the template. *NR1D1* expression levels in the different tissues were obtained by qPCR (Supplementary Figure S2). We found that *NR1D1* was expressed in all the yak tissues tested, and that the expression levels were highest in the muscle tissues (see Figure 1).

### 3.2 Establishment of a complete system of yak skeletal muscle satellite cell isolation

**Figure 2:**
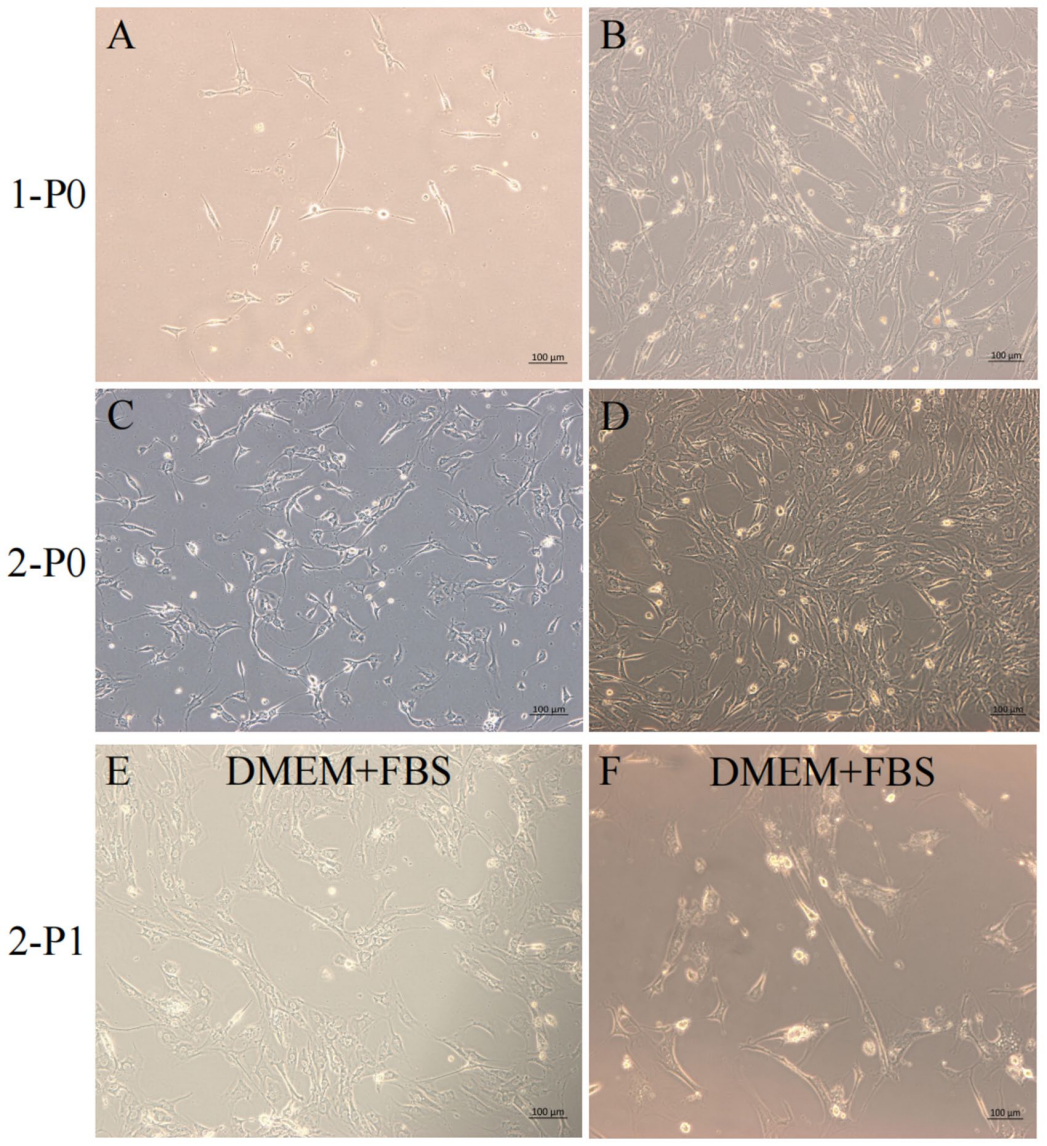
Yak SCs culture. (A–D) Primary culture of yak SCs, P0 generation cells, 1-P0 and 2-P0 are biological replicates. (E) P1 generation (2-P1) cells obtained after 2-P0 subculture and cultured in DMEM + FBS growth medium. (F) Cells cultured in DMEM + HS differentiation medium to induce cell differentiation. DMEM, Dulbecco’s modified Eagle’s medium; FBS, fetal bovine serum; HS, 2% horse serum.

**Figure 3:**
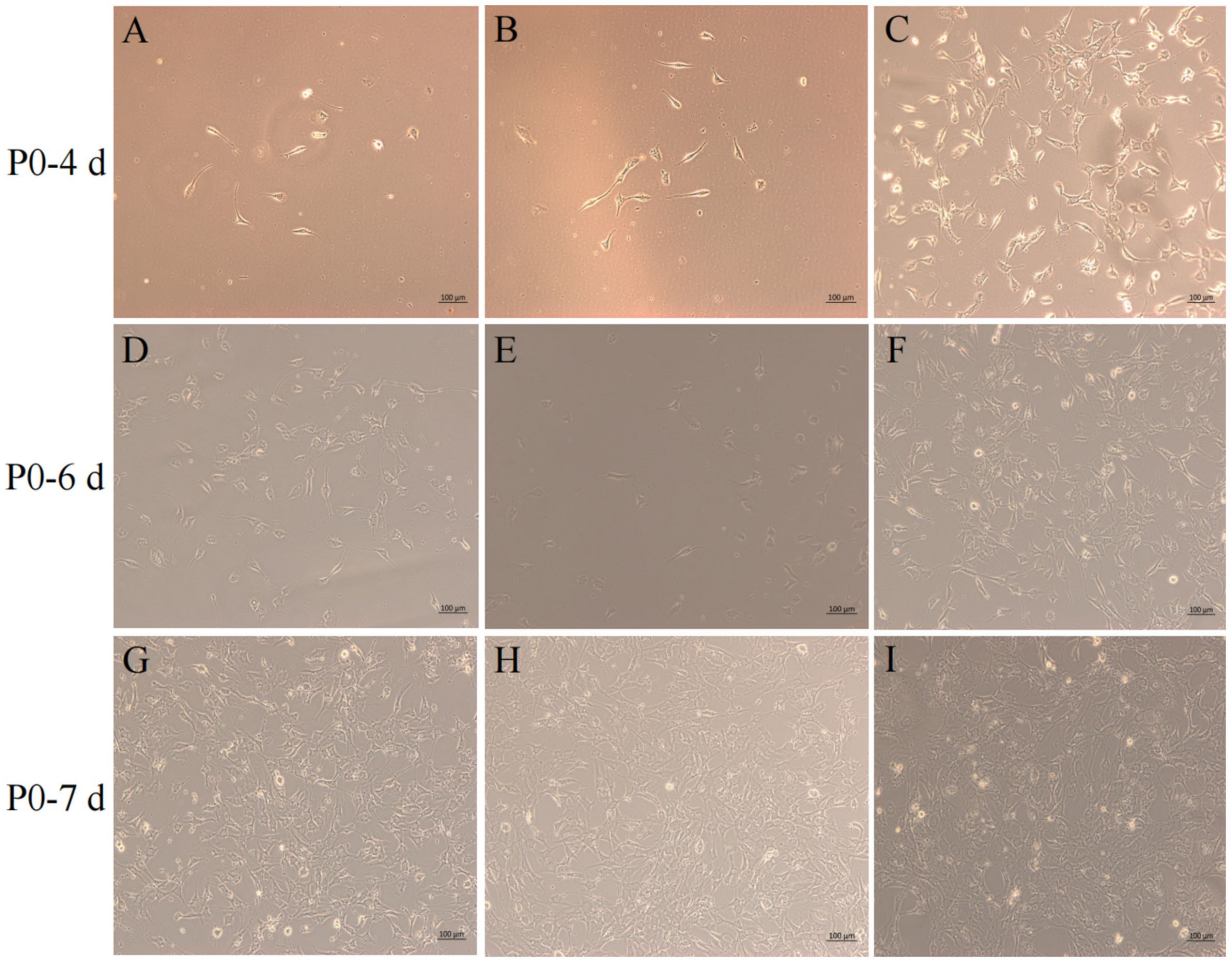
Screening of isolation conditions of yak SCs by cell separation. Yak SCs were separated by 40-μm and 70-μm cell sieves, and P0 cells were cultured in growth medium and mapped on days 4, 6, and 7. (A–C) Images taken on day 4. (D–F) Images taken on day 6. (G–I) Images taken on day 7.

**Figure 4:**
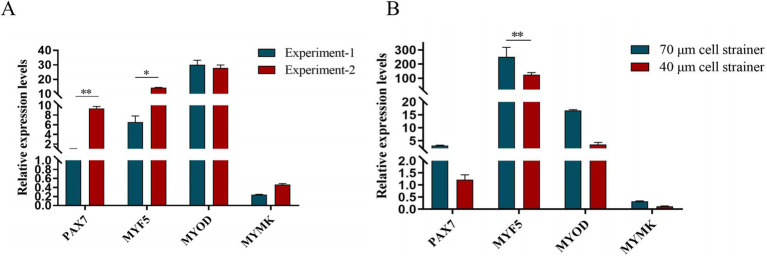
Screening of isolation conditions of yak SCs by marker gene expression levels. (A) Expression levels of myogenic gene mRNA in yak SCs isolated and cultured by two independent experiments. (B) Expression levels of myogenic gene mRNA in yak SCs filtered by 70-μm and 40-μm cell sieves. ^*^p < 0.05 and ^**^p < 0.01.

After isolation, we found that most of the primary yak SCs were long fusiform, a few were star, the middle of the cells were raised, and the refractive index was high (Figure 2). Muscle tissue contains various cell types, such as fibroblasts, blood cells, epithelial cells, and endothelial cells (22). Therefore, without purification, the proportion of SCs and the amount of muscle tube fusion are reduced. We determined the cell sieve pore size and purification process of yak SCs by sieve size and differential adhesion times. The cell separation method used in this study found no significant difference in the morphology of SCs obtained by 40-μm and 70-μm cell sieves; however, more cells were isolated by the 70-μm cell sieve (Figure 3). The mRNA expression levels the myogenic genes *PAX7*, *MYF5*, *MYOD*, and *MYMK* were analyzed by qPCR. The results showed that the expression levels of these genes were elevated in cells isolated using the 70-μm sieve compared to those isolated with the 40-μm sieve. Among them, a significant difference was observed in the *MYF5* expression level specifically between cells separated by the 40-μm and 70-μm sieves (Figure 4).

**Figure 5:**
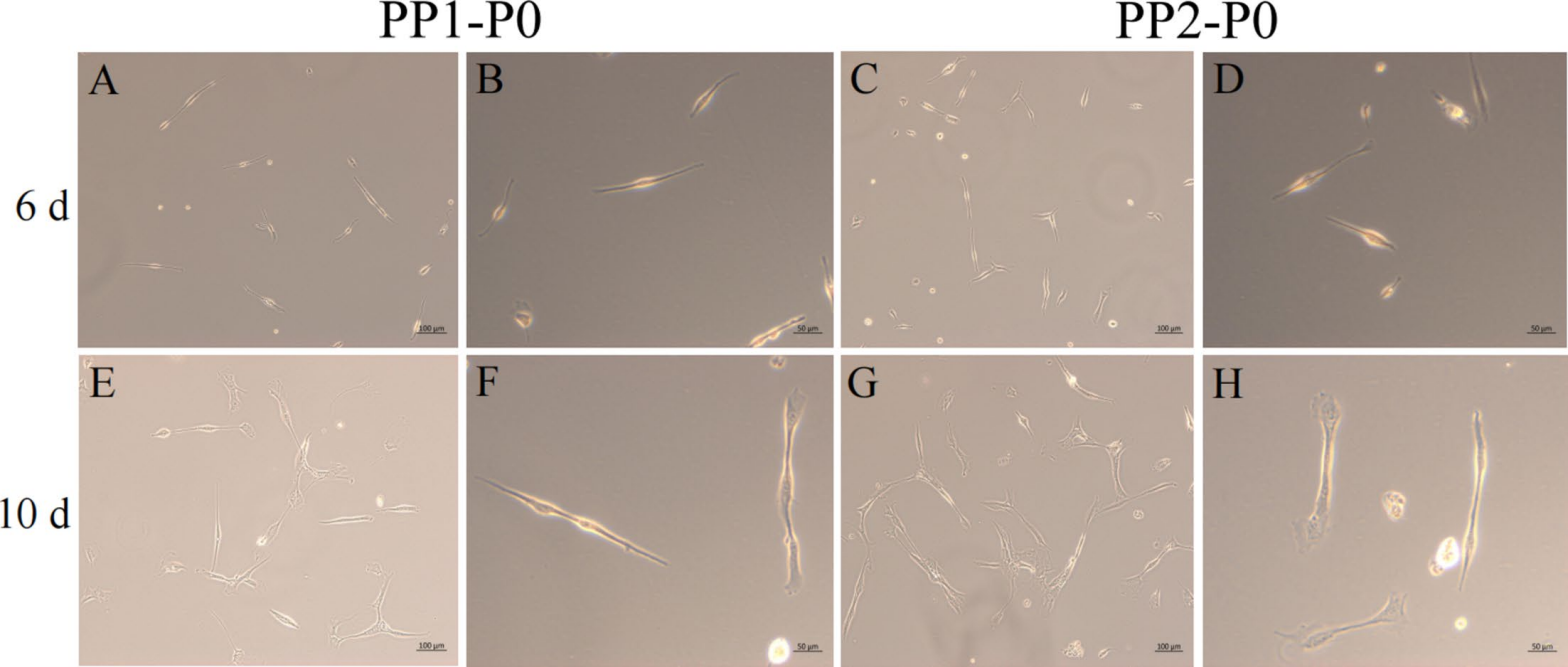
Yak SCs purification. P0 generation yak SCs by differential adhesion for 20 min, the unadherent cells were named PP1-P0, the unadherent cells were further cultured in walling culture for 1 h. The unadherent cells after this time were named PP2-P0. The cells were cultured in the growth medium and photographed on days 6 and 10. (A–D) Images taken on day 6. (E–H) Images taken on day 10.

**Figure 6:**
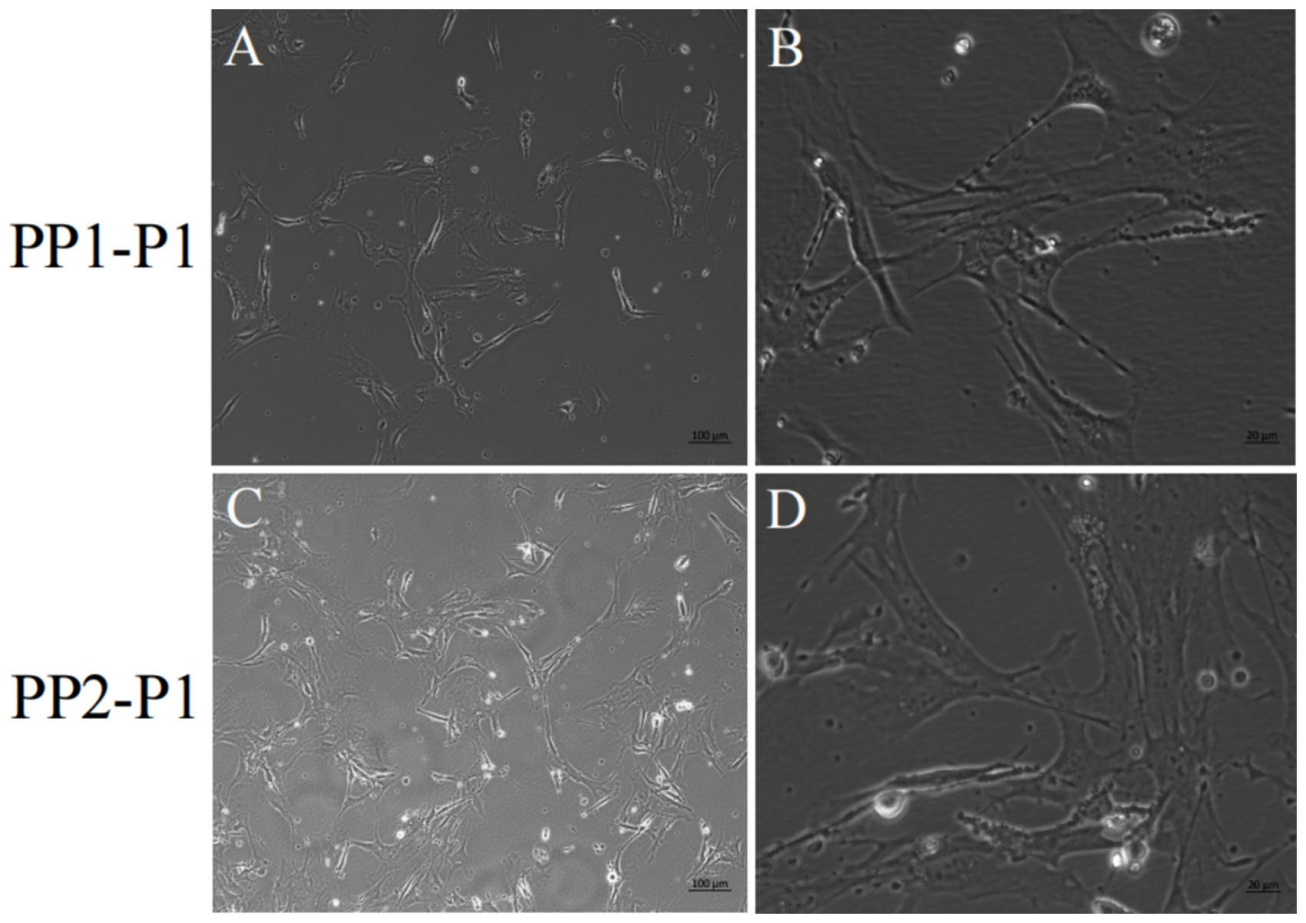
Yak SCs were purified and subcultured. (A,B) First generation yak SCs PP1-P1 after PP1-P0 subculture. (C,D) First generation yak SCs PP2-P1 after PP2-P0 subculture.

**Figure 7:**
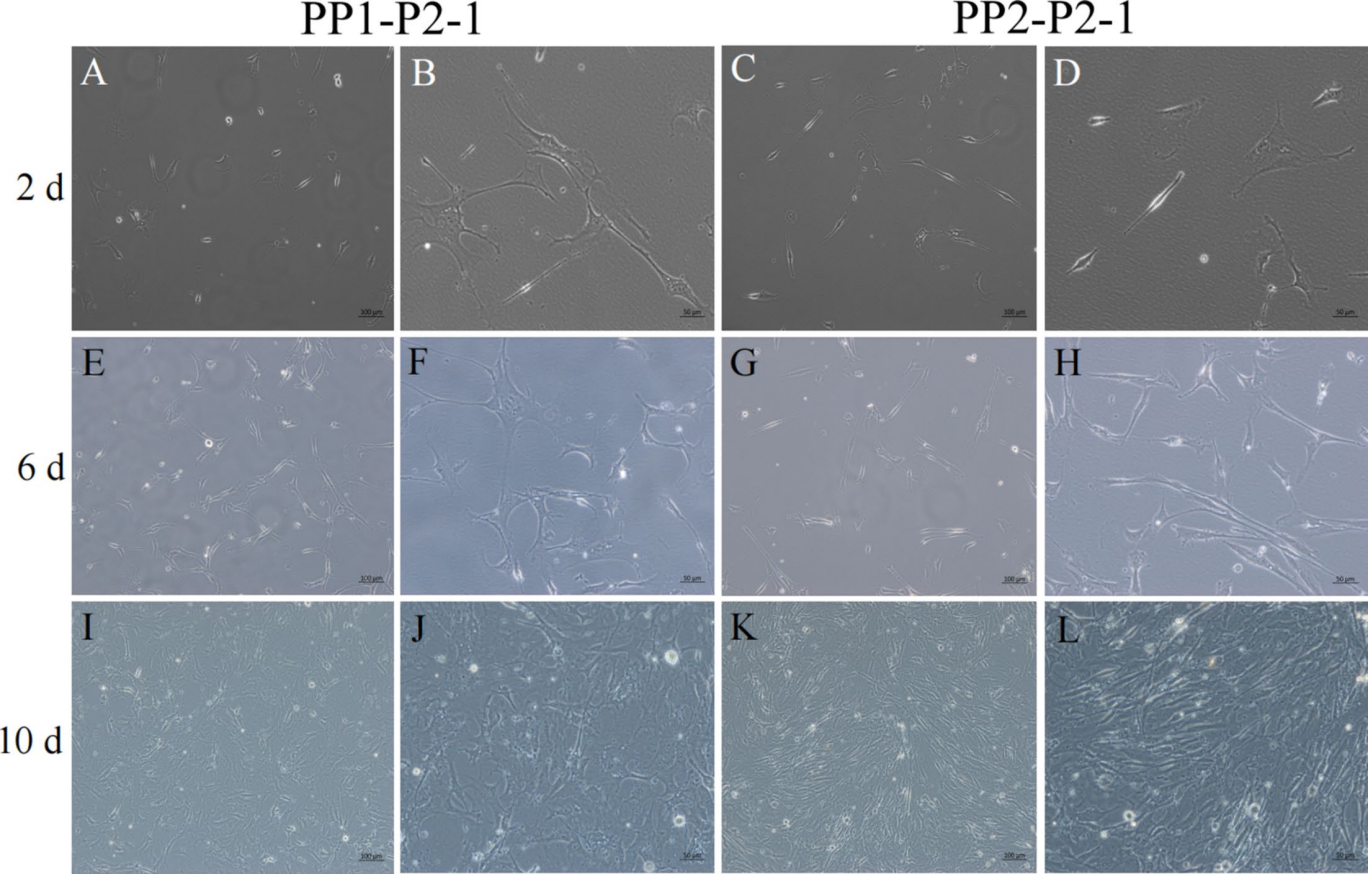
Yak SCs proliferated and cultured after subculture. PP1-P2-1 and PP2-P2-1 were cultured in growth medium and photographed on days 2, 6, and 10. (A–D) Images taken on day 2. (E–H) Images taken on day 6. (I–L) Images taken on day 10.

**Figure 8:**
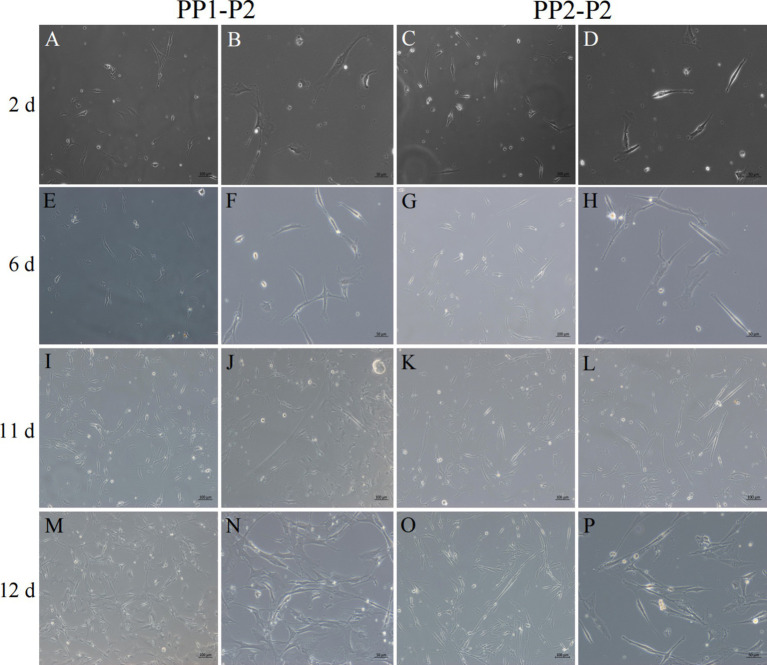
Isolation and culture of yak SCs subsets. After culture of PP1-P2-0 and PP1-P2-1 for 2 h, unadherent cells were PP1-P2. After culture of PP2-P1-0 and PP2-P2-1 for 2 h, unadherent cells were PP2-P2. Then, the PP1-P2 and PP2-P2 cells were cultured in growth medium and photographed on days 2, 6, 11, and 12. (A–D) Images taken on day 2. (E–H) Images taken on day 6. (I–L) Images taken on day 11. (M–P) Images taken on day 12.

**Figure 9:**
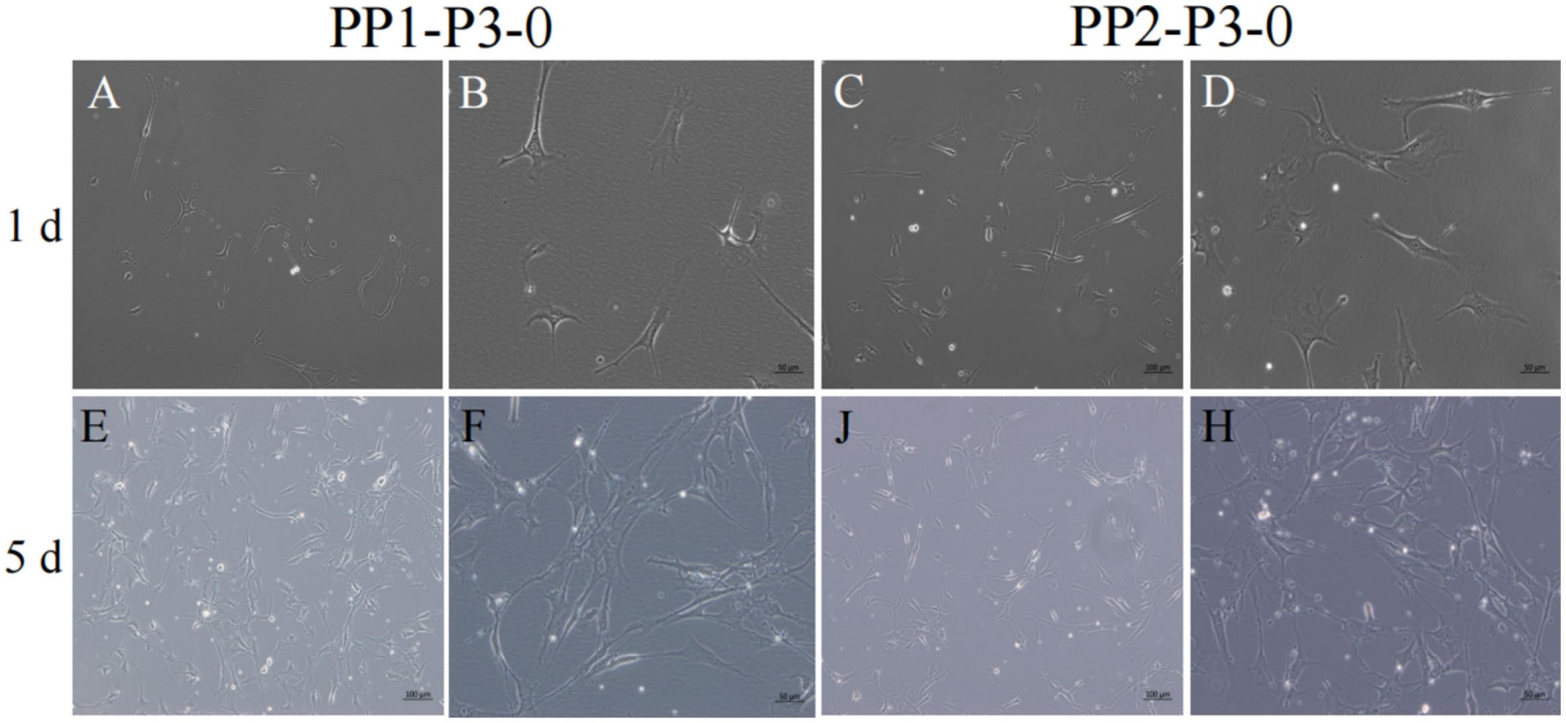
Subculture and culture of yak SCs. After the subculture of PP1-P2-0 and PP2-P2-0, the adherent cells were PP1-P3-0 and PP2-P3-0 after 15 min differential adhesion. The cells were cultured in the growth medium and photographed on days 1 and 5. (A–D) Images taken on day 1. (E–H) Images taken on day 5.

**Figure 10:**
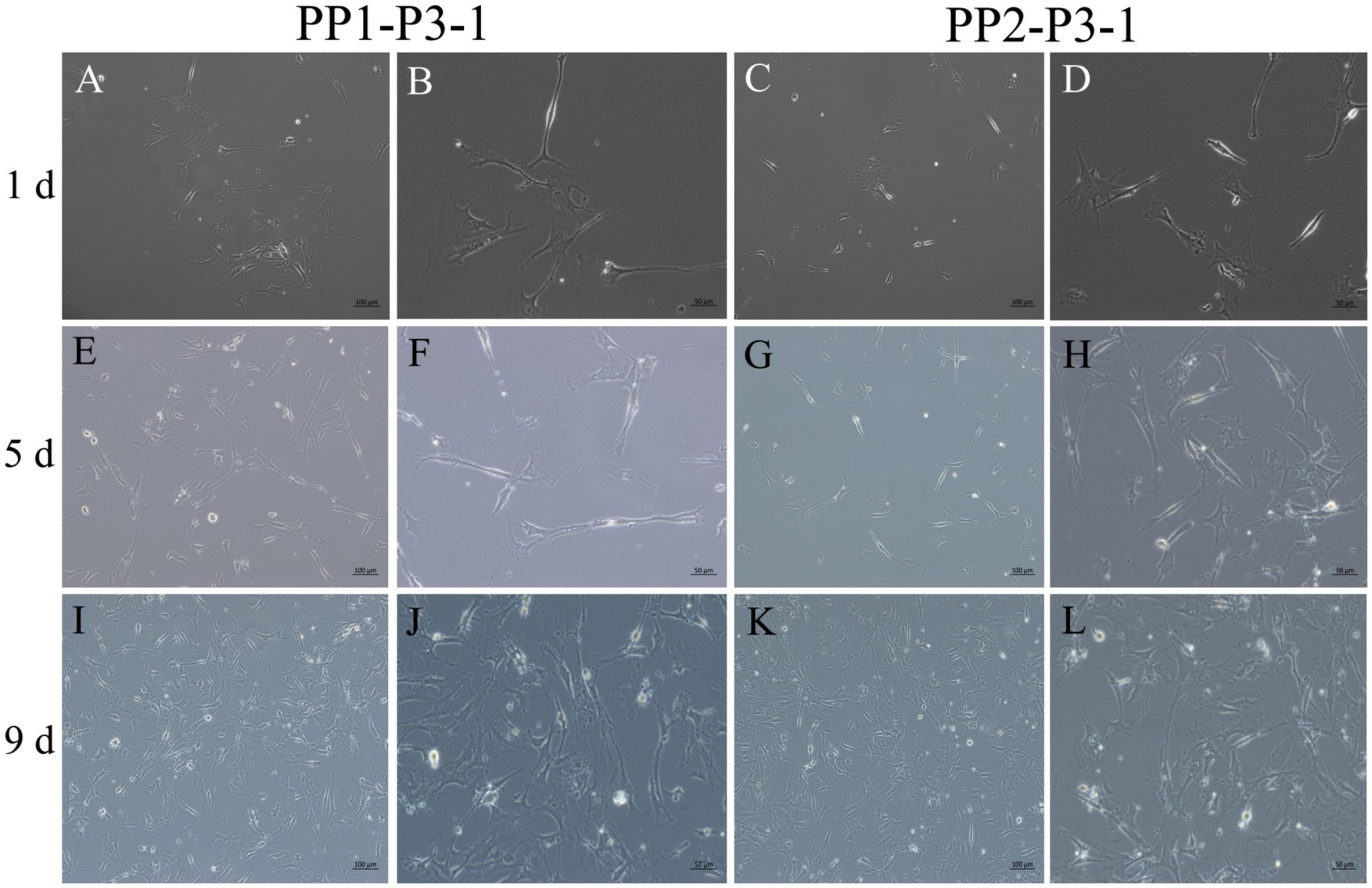
Subculture and culture of yak SCs. After the subculture of PP1-P2-0 and PP2-P2-0, the unadherent cells were PP1-P3-1 and PP2-P3-1. These cells were cultured in the growth medium and photographed on days 1, 5 and 9. (A–D) Images taken on day 1. (E–H) Images taken on day 5. (I–L) Images taken on day 9.

**Figure 11:**
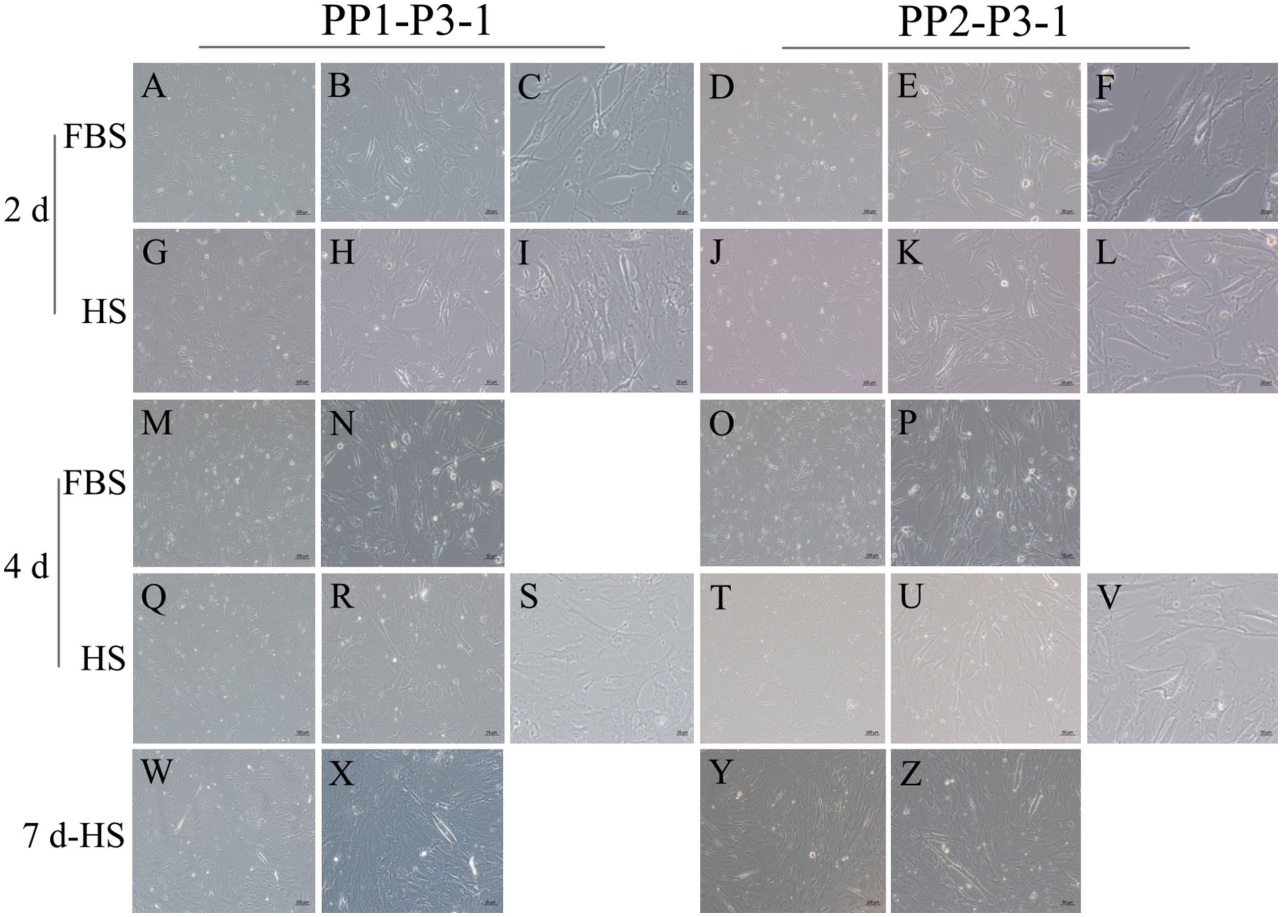
Proliferation and myogenic differentiation of third generation (P3) yak SCs. Myogenic differentiation of PP1-P3-1 and PP2-P3-1 cells was induced by DMEM + HS differentiation medium. The proliferation cells in DMEM + FBS growth medium were used as a control. The cells were photographed on days 2, 4, and 7 of culture. (A–C,G–I) Images of PP1-P3-1 taken on day 2. (D–F,J–L) Images of PP2-P3-1 taken on day 2. (M,N,Q–S) Images of PP1-P3-1 taken on day 4. (O,P,T–V) Images of PP2-P3-1 taken on day 4. (W,X) Images of PP1-P3-1 taken on day 7. (Y,Z) Images of PP2-P3-1 taken on day 7. DMEM, Dulbecco’s modified Eagle’s medium; FBS, fetal bovine serum; HS, 2% horse serum.

**Figure 12:**
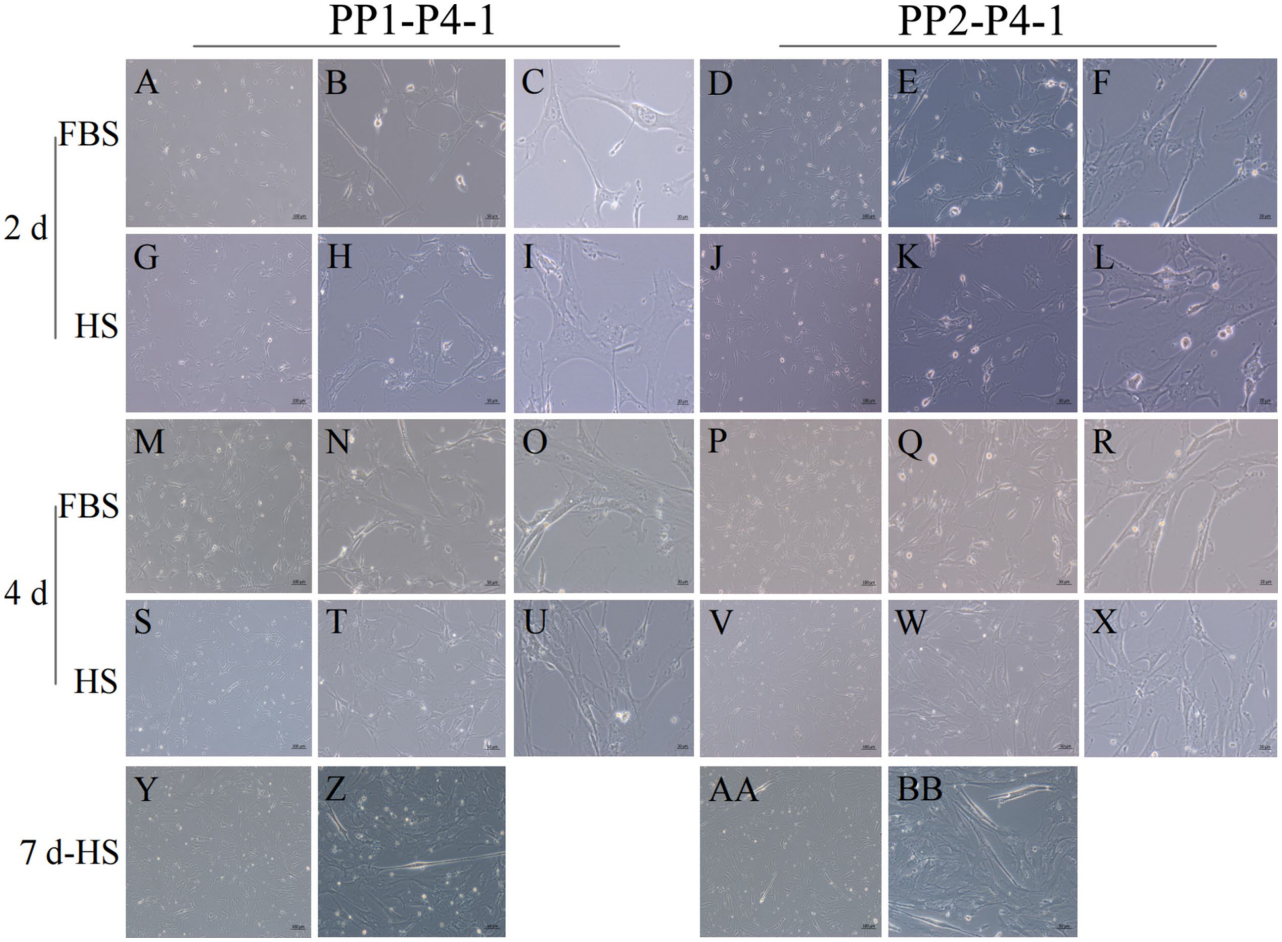
Proliferation and myogenic differentiation of forth generation (P4) yak SCs. Myogenic differentiation of PP1-P4-1 and PP2-P4-1 cells was induced by DMEM + HS differentiation medium. The proliferation cells in DMEM + FBS growth medium were used as a control. The cells were photographed on days 2, 4, and 7 of culture. (A–C,G–I) Images of PP1-P4-1 taken on day 2. (D–F,J–L) Images of PP2-P4-1 taken on day 2. (M–O,S–U) Images of PP1-P4-1 taken on day 4. (P–R,V–X) Images of PP2-P4-1 taken on day 4. (Y,Z) Images of PP1-P4-1 taken on day 7. (AA,BB) Images of PP2-P4-1 taken on day 7. DMEM, Dulbecco’s modified Eagle’s medium; FBS, fetal bovine serum; HS, 2% horse serum.

**Figure 13:**
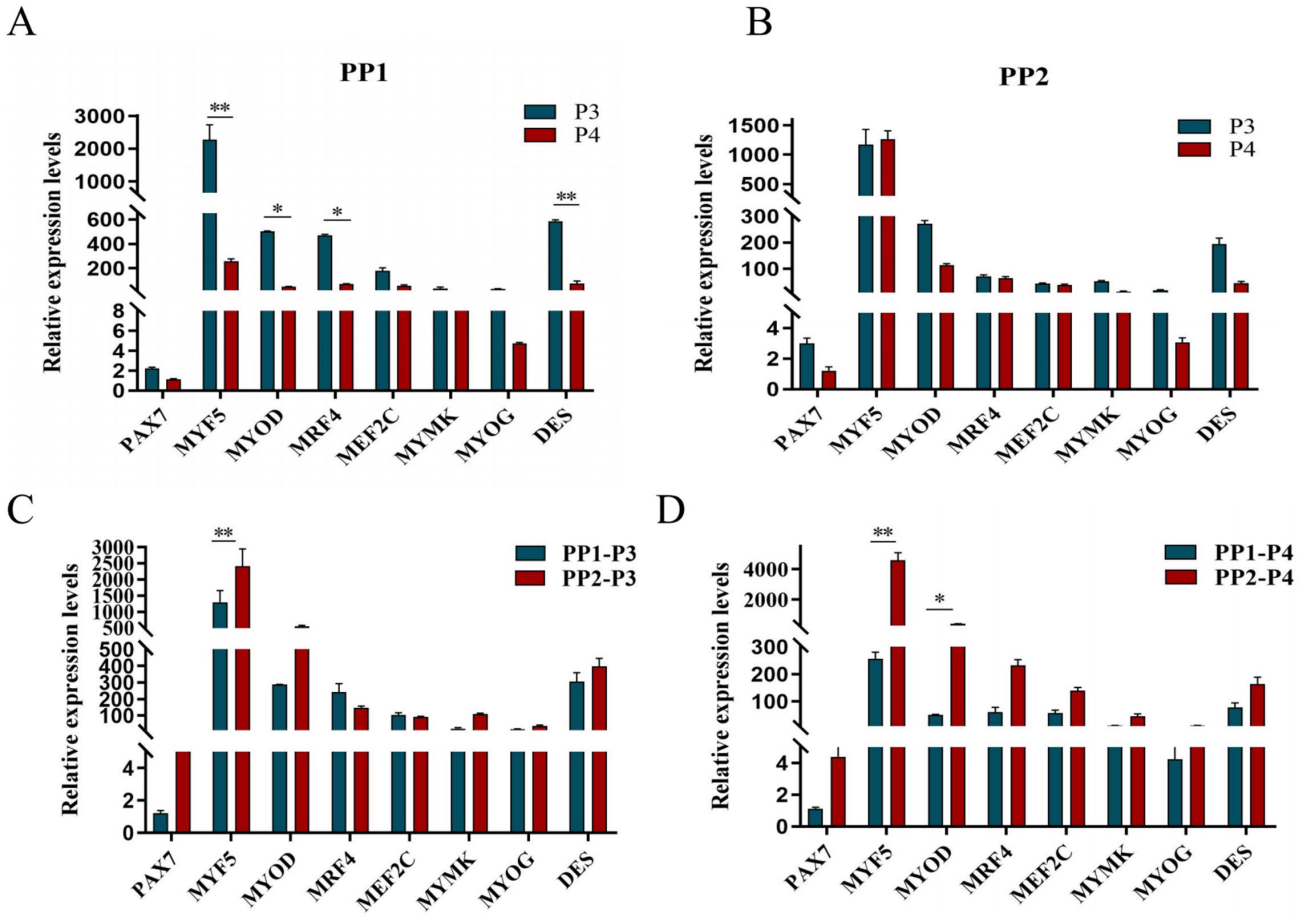
Purification and subculture of yak SCs. Yak SCs isolated from the skeletal muscle of yaks were purified by differential adhesion for 1 h; the adherent cells were named PP1 and the unadherent cells were PP2. Third (P3) and the fourth (P4) generation cells were cultured from the PP1 and PP2 cells. (A) Expression levels of myogenic gene mRNA in P3 and P4 cells of PP1. (B) Expression levels of myogenic gene mRNA in P3 and P4 cells of PP2. (C) Expression levels of myogenic gene mRNA in PP1-P3 and PP2-P3 cells. (D) Expression levels of myogenic gene mRNA in PP1-P4 and PP2-P4 cells. ^*^p < 0.05 and ^**^p < 0.01.

After continuous differential adhesion and subculture, the third (P3) and fourth (P4) generation of PP1 and PP2 (yak SCs isolated from the skeletal muscle of yaks were purified by differential adhesion for 1 h; the adherent cells were named PP1 and the unadherent cells were PP2) were obtained. The PP2 subset was purified once more than the PP1 subset. We found that the P0 generation yak SCs were round and had strong refraction. With the increase of adhesion time, the shape changed from round to fusiform and spindle shape, and the density gradually increased, indicating that the isolated and purified SCs had strong proliferation ability (Figures 5–12; Supplementary Figure S3). Compared to P3 cells, the expression levels of myogenic genes *MYF5*, *MYOD*, *MRF4*, and *DES* in the P4 cells of PP1were significantly reduced (Figure 13A). In the P3 and P4 cells of PP2, there were no significant differences in the expression of these four genes (Figure 13B). The expression of *MYF5* in PP2-P3 cells was significantly higher than its expression in PP1-P3 cells, indicating a significant difference between the two cell types (Figure 13C), and the expression levels of *MYF5* and *MYOD* in PP2-P4 cells were significantly higher than their expression levels in PP1-P4 cells (Figure 13D). The above results indicate that there may be certain differences in the cell types and proportions of SCs contained in PP1 and PP2.

**Figure 14:**
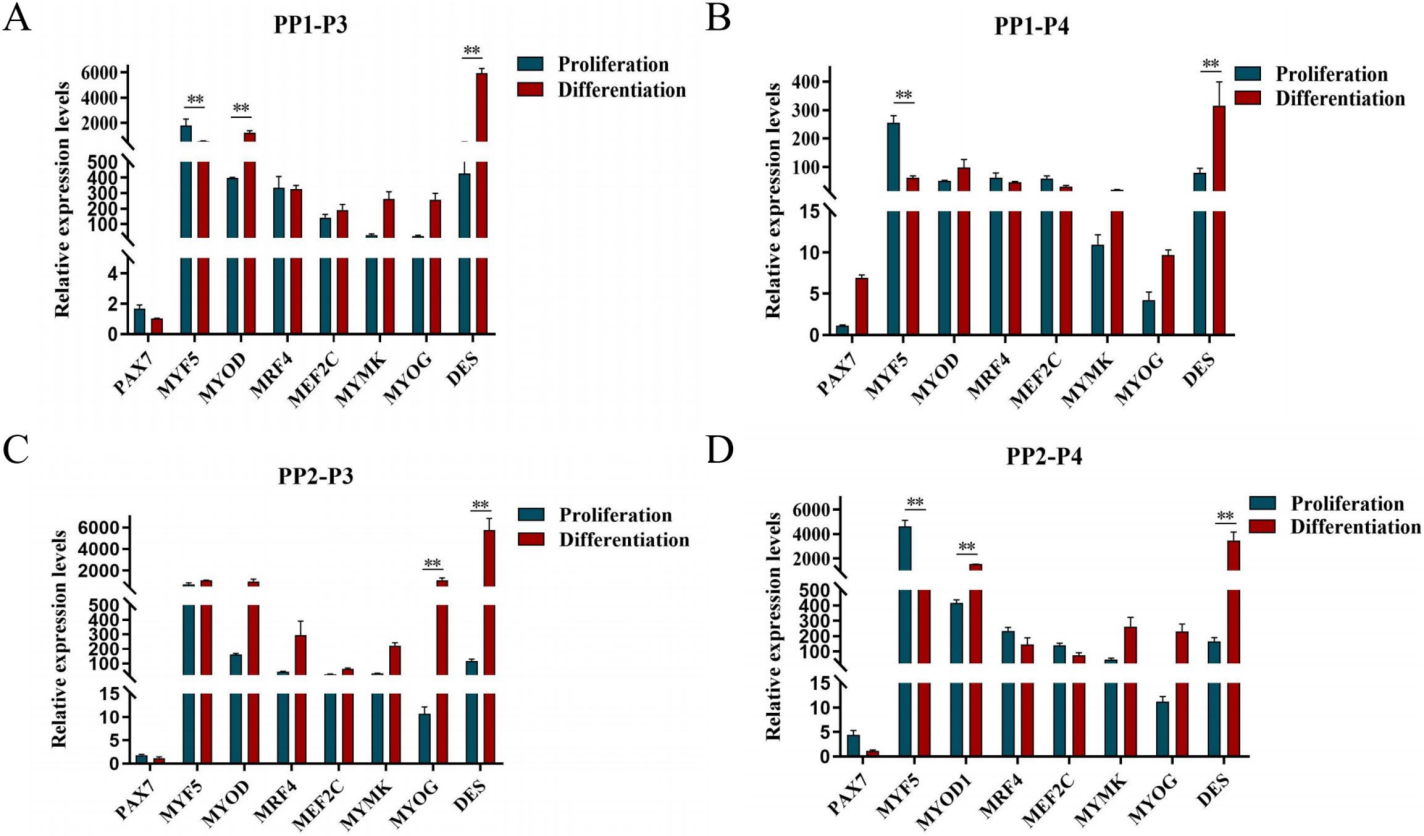
Proliferation and differentiation of yak SCs. Third (P3) and forth (P4) generations of yak SCs were multiplied and differentiated in DMEM + FBS growth medium and DMEM + HS differentiation medium. (A) Expression levels of myogenic gene mRNA in PP1-P3 cells. (B) Expression levels of myogenic gene mRNA in PP1-P4 cells. (C) Expression levels of myogenic gene mRNA in PP2-P3 cells. (D) Expression levels of myogenic gene mRNA in PP2-P4 cells. DMEM, Dulbecco’s modified Eagle’s medium; FBS, fetal bovine serum; HS, 2% horse serum; ^*^p < 0.05 and ^**^p < 0.01.

After induction of differentiation, the expression of *MYF5* in all cells except PP2-P3 decreased significantly, while the expression of *DES* increased significantly. *MYOD* expression increased significantly in both PP1-P3 and PP2-P4, and *MYOG* expression increased significantly in PP2-P3 (Figure 14). The increase in *MYOD* expression after differentiation indicates that the myogenic gene program is gradually activated.

### 3.3 *NR1D1* inhibits proliferation and differentiation of SCs

**Figure 15:**
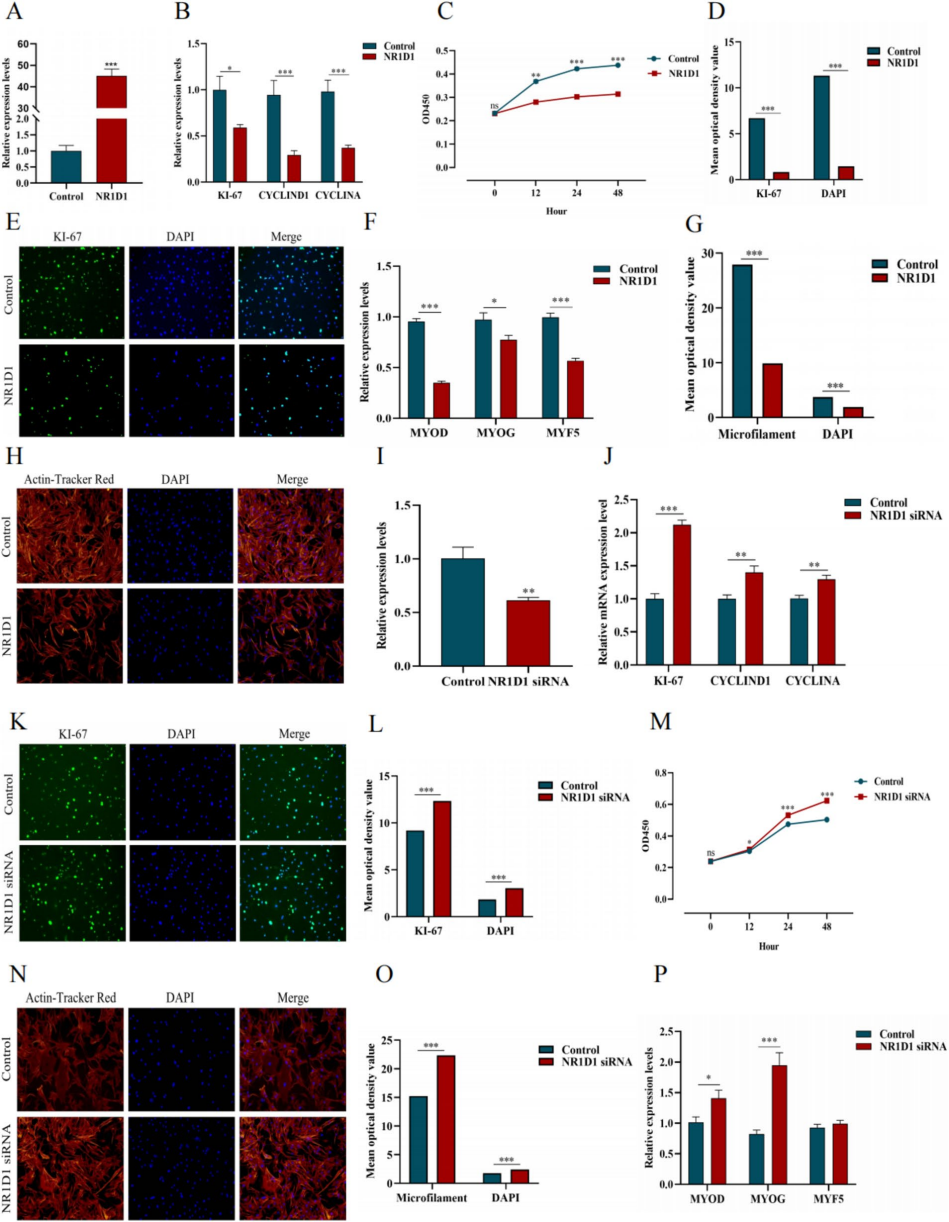
Effects of *NR1D1* on proliferation and differentiation of yak SCs. (A) Overexpression efficiency of *NR1D1*. (B) Expression of proliferation marker genes after *NR1D1* overexpression. (C) CCK-8 measurement after *NR1D1* overexpression. (D) The average optical density value after *NR1D1* overexpression. (E) Fluorescence specific staining after *NR1D1* overexpression. (F) Expression of differentiation marker genes after overexpression of *NR1D1*. (G) The average optical density value after *NR1D1* overexpression. (H) Fluorescence specific staining after *NR1D1* overexpression. (I) Interference efficiency of *NR1D1*. (J) The expression of proliferation marker genes after *NR1D1* interference. (K) Fluorescence specific staining after *NR1D1* interference. (L) The average optical density value after *NR1D1* interference. (M) CCK-8 measurement after *NR1D1* interference. (N) Fluorescence specific staining after *NR1D1* interference. (O) The average optical density value after *NR1D1* interference. (P) *NR1D1* interferes with the expression of differentiation marker genes. Control indicates the respective controls, ^*^p < 0.05, ^**^p < 0.01, and ^***^p < 0.001.

We constructed the *NR1D1* overexpression vector and achieved good transfection efficiency (Figure 15A). Overexpression of *NR1D1* significantly reduced the expression levels of proliferation marker genes (Figure 15B). CCK-8 detection and specific fluorescence staining (We have conducted a semi-quantitative statistical analysis of stained cells or microfilaments, the same below) (Figures 15C–E) further confirmed that *NR1D1* inhibited the proliferation of satellite cells. Subsequently, the detection of differentiation marker genes and the specific staining of microfilament red fluorescent probes (Figures 15F–H) showed that the overexpression of *NR1D1* significantly decreased the levels of differentiation marker genes and inhibited the number of microfilaments. Next, we designed a siRNA targeting *NR1D1* to reduce its expression (Figure 15I). After knocking down *NR1D1*, the proliferation marker gene increased significantly (Figure 15J), and specific fluorescence staining and CCK-8 detection (Figures 15K–M) also showed that knocking down *NR1D1* significantly promoted cell proliferation. The results of differentiation marker gene detection and specific staining with microfilament red fluorescent probes (Figures 15N–P) showed that the expression level of differentiation marker genes increased and the number of microfilaments increased. These findings contrast with the results of *NR1D1* overexpression, suggesting that *NR1D1* plays an important role in inhibiting the proliferation and differentiation of satellite cells.

## 4 Discussions

### 4.1 Complete system of yak SCs isolation and culture was established

*PAX7* is a unique marker gene for satellite cells during both the quiescent and proliferative phases (23–25). In the quiescent phase, *PAX7* is expressed in satellite cells (26); during proliferation, the most prominent myogenic factors expressed are *MYF5* and *MYOD*. After proliferation, most cells begin to downregulate their *PAX7* expression and maintain *MYOD* for differentiation, while the remaining cells maintain *PAX7* expression and suppress *MYOD* to return to a quiescent state. When satellite cells commence differentiation and fuse into myotubes, *MYOG* expression increases (27). In this study, we examined the mRNA expression levels of specific marker genes in satellite cells at different stages and found that the gene expression trends were largely consistent with the above descriptions. Since muscle tissue comprises multiple cell types, purification steps are crucial. We determined the yak satellite cell purification process through differential adhesion times. From the cell images, it can be observed that there are significant differences in the proliferation levels of PP1 and PP2 cells and their respective passaged cells, as well as in the fusion levels of differentiated cells. In terms of relative gene expression, the decline in skeletal muscle-derived genes among passaged PP2 cells is not significant, while the expression levels of *MYF5* and *MYOD* are significantly higher than those in PP1 cells, which is consistent with the above statement and in sharp contrast to the significant decrease in *MYF5*, *MYOD*, *MRF4*, and *DES* expression after passaging in PP1 cells. This indicates that there are differences in the types and proportions of purified cells. At the level of myogenic regulatory factors, the gradual downregulation of *PAX7* expression and enhanced *MYOD* expression serve as markers for the transition of satellite cells from proliferation to differentiation (28). If stem cells maintain *PAX7* expression and suppress *MYOD* levels, the differentiation process is hindered. However, once muscle satellite cells express *MYOG*, *PAX7* expression in these cells is inhibited, and differentiation subsequently occurs, suggesting an inter-inhibitory mechanism between *MRFs* and *PAX7*. In this study, after inducing satellite cell differentiation, we found that *MYF5* expression showed a significant downward trend, while *MYOD* expression gradually increased after differentiation culture, indicating the gradual activation of the myogenic gene program. *MYOG* and *DES* are indicators of terminal muscle differentiation, with *MYOG* being an essential protein for the formation of new muscle fibers. Similarly, we also found that *MYOG* and *DES* expression significantly increased after satellite cell differentiation. With the proficiency of separation technology, we conducted another independent replication experiment-2. qPCR verification (Figure 4A) showed that the purity and accuracy of the isolated cells had been improved compared to previous results. In summary, these findings demonstrate that we have successfully established a relatively complete system for the isolation and culture of yak satellite cells.

### 4.2 *NR1D1* inhibits proliferation and differentiation of SCs to a certain extent

Yak meat is characterized by low fat and high protein, is rich in nutrients such as essential amino acids, fatty acids, vitamins, and minerals (29), and is the main source of animal protein for residents in the Qinghai-Tibet Plateau. Because of the adverse environment in the high altitude area, yaks grow slowly and have problems such as muscle retardation, poor fat deposition efficiency, and poor tenderness of muscle tissue, which limits the market development of yaks (30). Skeletal muscle is the most economically valuable tissue of meat-producing animals, and its yield and quality directly determine the productivity benefit of animals. SCs contribute to skeletal muscle fiber hypertrophy and the mutual transformation of skeletal muscle fiber types by proliferating, differentiating, and fusing with muscle fibers to form new myonuclei, thereby influencing meat quality (31).

During skeletal muscle growth and development, a series of regulatory changes occur in gene expression (32–35), and deciphering these changes is crucial for the production of high-quality meat products (36). There is increasing evidence that the biological clock is crucial for maintaining skeletal muscle mass and growth (37). The circadian clock circuit, consisting of an interlocking molecular network of transcriptional and translational regulatory factors, is involved in various aspects of skeletal muscle function (38, 39), ranging from oxidative metabolism, sarcomere structure, contractile properties to muscle mass maintenance. As a ligand-dependent nuclear receptor (21, 40–42). *NR1D1* is a key inhibitor in the molecular clock transcriptional network (43). *NR1D1* was found to be involved in many aspects of metabolism shortly after its discovery, including adipocyte differentiation (44) and muscle formation (45). In adult mice, *NR1D1* mRNA is universally expressed with the most abundant levels found in brown fat, skeletal muscle, and brain (10, 46). Pircher et al. (47) studied *NR1D1* in native skeletal muscle and found that in mice lacking *NR1D1*, specific fiber types were preferentially expressed and there were differences in the composition of muscle fiber types. Chatterjee et al. (43) also found that the loss of *NR1D1* accelerated the morphological transformation of primary myoblasts into organized muscle tubes, the number of myosin heavy chain muscle fibers increased, and the percentage of fused muscle fibers containing multiple nuclei was high. In this study, we examined the expression abundance of *NR1D1* in different tissues through qPCR and found that *NR1D1* was most highly expressed in muscle tissue. In addition, based on the results of qPCR, CCK8, and specific fluorescence staining, as well as semi-quantitative analysis of KI-67, DAPI-stained cells and microfilaments, we found that *NR1D1* inhibited the proliferation and differentiation of yak satellite muscle cells to a certain extent. Chatterjee et al. (43) also found that *NR1D1* inhibited of myogenic precursor cell proliferation and myogenic properties, which inhibited skeletal muscle regeneration *in vivo*. Notably, the myogenic regulatory factors gene family includes *MYOD*, *MYF5*, *MYOG*, and *MRF4*, all of which are important factors in the process of myocytogenesis with roles in the development and genesis of muscle, as well as in guiding the specialization of muscle satellite cells and skeletal muscle regeneration. *MYOD* and *MYOG* in particular contribute greatly to the regulation of skeletal muscle development. They have been shown to control muscle development by regulating myoblast proliferation and phagocytosis, and to influence skeletal muscle formation by promoting multinuclear muscle tube formation and muscle fiber maintenance. Interestingly, *MYOD* was better than *MYF5* in inducing myoblast differentiation and expression, whereas *MYF5* was more efficient than *MYOD* in promoting myoblast proliferation. Maintaining proper myogenic precursor cell proliferation and differentiation function is essential for skeletal muscle growth and repair (48, 49).

To explore how *NR1D1* regulates skeletal muscle, we have mapped out the regulatory pathway of *NR1D1*’s influence on muscle development (Supplementary Figure S4). *NR1D1* may potentially modulate the proliferation, differentiation, and growth of skeletal muscle satellite cells through this mechanism. The Wnt pathway serves as a primary developmental signal driving muscle development and repair. β-catenin, a key signal transducer in the classical Wnt signaling pathway, responds to Wnt ligands and accumulates in the cytoplasm, ultimately leading to its nuclear translocation and activation of Wnt target genes, which in turn regulate cell cycle progression. Bmal1, an essential transcriptional regulator in the circadian transcriptional feedback loop, can dimerize with Clock to activate downstream target genes such as Period genes (*Per1*, *Per2*, and *Per3*) and Cryptochrome genes (*Cry1* and *Cry2*), which subsequently inhibit the activity of Bmal1/Clock. Within myoblasts, Bmal1 acts as a regulator in the classical Wnt signaling pathway, controlling components including the ligand Wnt10a, the signal transduction mediator Dishevelled 2 (*Dvl2*), β-catenin, the transcription factor T-cell factor 3 (*TCF3*), and the Wnt pathway target gene *Axin2*. As a crucial inhibitory factor in the molecular clock transcriptional network, *NR1D1* inhibits Bmal1 transcription by binding to Rore, while Rore can activate Bmal1 gene transcription. This antagonistic regulation gives rise to *Bmal1*’s circadian oscillations. Furthermore, *NR1D1* may also regulate different steps within the Wnt pathway.

### 4.3 *NR1D1* affects low temperature adaptation

Studies on an adipogenic cell line showed that *NR1D1* was essential for adipocyte differentiation (49–51). Recently, *NR1D1* was found to be active in brown adipose tissue (BAT) (52), which is the main site of heat production in the body (53). Mice lacking *NR1D1* reduced the lowest point of temperature oscillation by disinhibition of uncoupling protein 1 (UCP1), which is the direct target of *NR1D1* in brown adipose tissue (52). *NR1D1* has also been shown to play a key role in protecting body temperature from cold challenges, so mice that are genetically deficient in *NR1D1* or at the low of *NR1D1* expression are protected from extreme cold. The environment in which yaks live has a long cold season and a short warm season, so understanding and applying the relevant regulatory mechanism of *NR1D1* may affect the degree of adaptability of yaks to cold environment.

### 4.4 Trend of *NR1D1* targeted therapy for cancer

The circadian clock imposes daily rhythms in cell proliferation, metabolism, inflammation, and DNA damage responses, and perturbations of these processes are hallmarks of cancer, Furthermore, chronic circadian disturbance predisposes individuals to tumors. Therefore, the hypothesis that pharmacological regulation of circadian mechanisms could be an effective therapeutic strategy against cancer seems reasonable. In recent years, various cancer targeting strategies related to *NR1D1* have been introduced. For example, Ka et al. (54) found that cancer cells showed elevated levels of intracellular reactive oxygen species (ROS) due to accelerated metabolism, mitochondrial dysfunction, and antioxidant deficiency. *NR1D1* inhibits the DNA repair of ROS-induced DNA damage in breast cancer cells and enhances the accumulation of DNA damage, thereby increasing the sensitivity of breast cancer cells to oxidative stress. Thus, *NR1D1* may be a therapeutic target for breast cancer treatment, especially in patients treated with ROS-inducing chemotherapy drugs. Breast cancer is the most common cancer in women worldwide and the leading cause of cancer death. Current triple-negative breast cancer (TNBC) treatment is based on chemotherapy and radiation, which are associated with serious side effects. Therefore, identifying new targets may be beneficial for treating women with TNBC by minimizing side effects. Na et al. (55) showed that high *NR1D1* expression levels had favorable effects on overall survival and disease-free survival of patients with TNBC who were receiving chemotherapy. *NR1D1* should be further investigated as a possible prognostic marker for patients with TNBC receiving chemotherapy and as a target for the development of chemotherapy approaches to treat TNBC. Yang et al. (56) showed that patients with bladder cancer with positive *NR1D1* expression had longer disease-free survival than those with negative *NR1D1* expression. Cell viability, migration, and colony formation of bladder cancer cells were significantly inhibited after treatment with *NR1D1* agonist SR9009. Thus, the overexpression of *NR1D1* inhibited the tumorigenicity of bladder cancer cells, and *NR1D1* played a role in cancer inhibition, implying it may be a new target for the treatment of bladder cancer. Kim et al. (57) showed that *NR1D1* acted as a tumor suppressor in a tumor microenvironment by negatively regulating the PYD domain-containing protein 3 (NLRP3) inflammasome, suggesting that blocking the NLRP3 inflammasome by *NR1D1* activation may be a therapeutic strategy to treat lung cancer. Together, *NR1D1* is a high-function transcriptional suppressor that has therapeutic potential in cancer therapy. The role of *NR1D1* in other biological functions needs to be further explored.

## 5 Conclusion

We isolated and identified yak SCs, established a comprehensive system for the isolation and culturing of yak muscle satellite cells, and investigated the effects of *NR1D1* on proliferation and differentiation of SCs. We found that *NR1D1* inhibited the proliferation and differentiation of SCs to some extent. Yak meat has rich nutritional value and is a green food that meets the demand of modern markets. The growth and development rate of skeletal muscle determines the meat production performance of livestock and poultry, and the composition of muscle fiber types is closely related to the quality of muscle. Our results have reference value for studying the growth and development of skeletal muscle and muscle fiber type composition of livestock and poultry, and have positive social and economic significance for increasing meat production performance and improving meat quality of livestock and poultry. Currently, there is a need for more in-depth research on the regulation of satellite cells by *NR1D1* in controlling muscle fiber characteristics, which in turn affects the meat quality of livestock and poultry. The aim is to improve meat quality to a certain extent and play a role in enhancing meat production yield and quality, thereby contributing to social and economic significance.

## Data Availability

The data presented in the study are deposited in the NCBI repository, accession number SRR29014858.
