# Supplementary material for: Effect of NR1D1 on the proliferation and differentiation of yak skeletal muscle satellite cells
Source: Front Vet Sci. 2024 Nov 4;11:1428117. doi: 10.3389/fvets.2024.1428117 (PMC11571325; doi:10.3389/fvets.2024.1428117)
Supplement: Supplementary file 1 [file Data_Sheet_1.pdf]

Supplementary Table S1. Sequence of primer

Gene	Forward primer (5'-3')	Reverse primer (5'-3')
PAX7-1	GCCGAGTGCTCAGAATCAAG	CGTCCAGACGGTTCCCTTT
MYF5-1	ACGATGGACATGATGGACGG	AAACTCGTCCCCGAACTCAC
MYOD-1	TCAGACCCTCAGTGCTTTGC	CGACAGCAGCTCCATATCCC
MYMK-1	GCCCCTGAATCAAGCCATCT	TGATGAAATGATGAGGGTGAACA
MRF4-1	ATTCCAGGGGGCTCGTGATA	ATCAATGCTTGTCCCTCCTTCC
MYOG-1	AGCAAGAGGGCAGTTCTGTT	CCTGACCAGAGGGCTTACAC
MEF2C-1	TCAGACACTTGAGCACACGC	TCAATCCAATAGCAGCCCGA
DES -1	AGCCGGATCAACCTCCCTAT	ACCTCATGCTGCTGTTGTGT
GAPDH-1	CTGCCCGTTCGACAGATAGC	GAATCCGTTCACTCCGACCT
NR1D1-1	CCAGCAAGAGCACCAGCAACAT	CGATTGATGCGGACGATGGAG
KI-67-1	GAGGTGGCTCAGGTTCGTC	AAAGGGTTGGTGGTAAGTGGC
CYCLIND1-1	GAAGTGCGAGGAAGAGGTCT	TAAATGCACAGCTTCTCGGC
CYCLINA	CCAGTCTATCGTAGGGGCAG	ACTGCACGATGACCTGGTTA
MYOG-1	GGCTGACAAATGCCAGACTATCC	TGGTCCCTTGCTTTATCTCCCT
MYF5-1	ACGATGGACATGATGGACGG	AAACTCGTCCCCGAACTCAC
NR1D1-2	cagcggtttaaacttaagcttATGACG ACCCTGGACTCTAACAAC	ccacactggactagtggatccGAGGG AGGCAGGTATTTACAAGAA
NR1D1 siRNA	AACAACAACACAGGTGGCGTC	ATGCTCCCAAAAGAGCGAGC

Note: 1: Quantitative Reverse-Transcription PCR primer; 2: Amplification primer of full-length sequence.
Lowercase letters indicate homologous arms .
The data presented in the study are deposited in the NCBI repository, accession number SRR29014858.

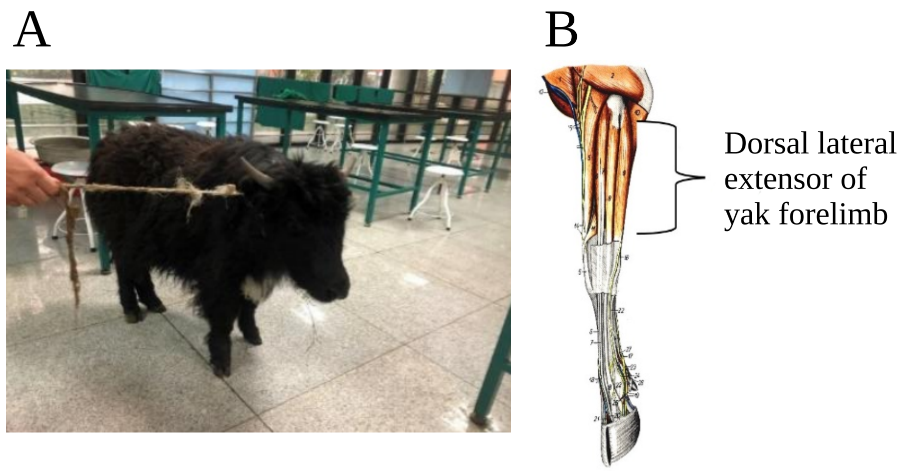

Supplementary Figure S1. Yak skeletal muscle sample. (A) Yak calf. (B) Yak forelimb skeletal muscle.

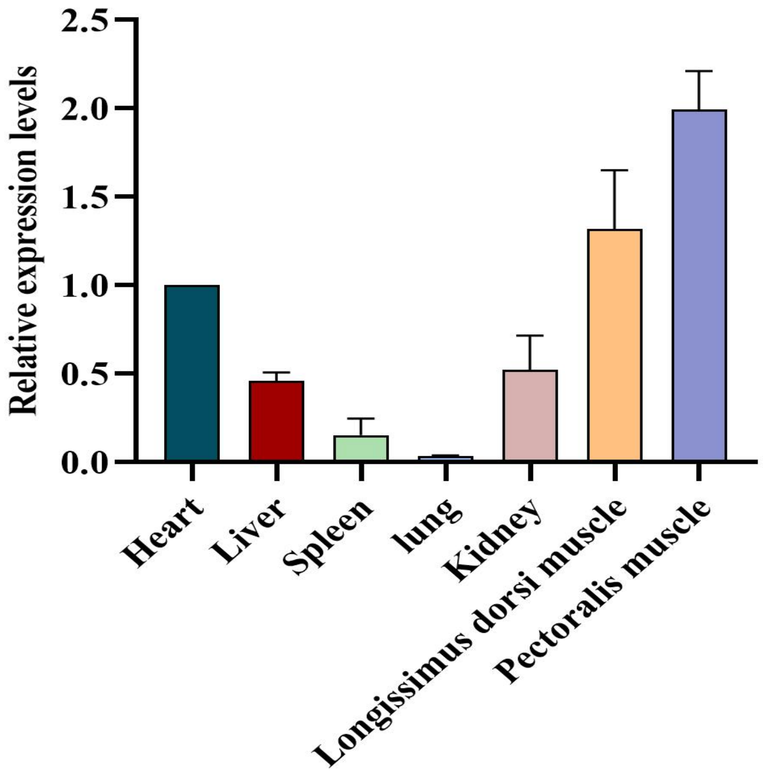

Supplementary Figure S2. Expression levels of *NR1D1* mRNA in different yak tissues.

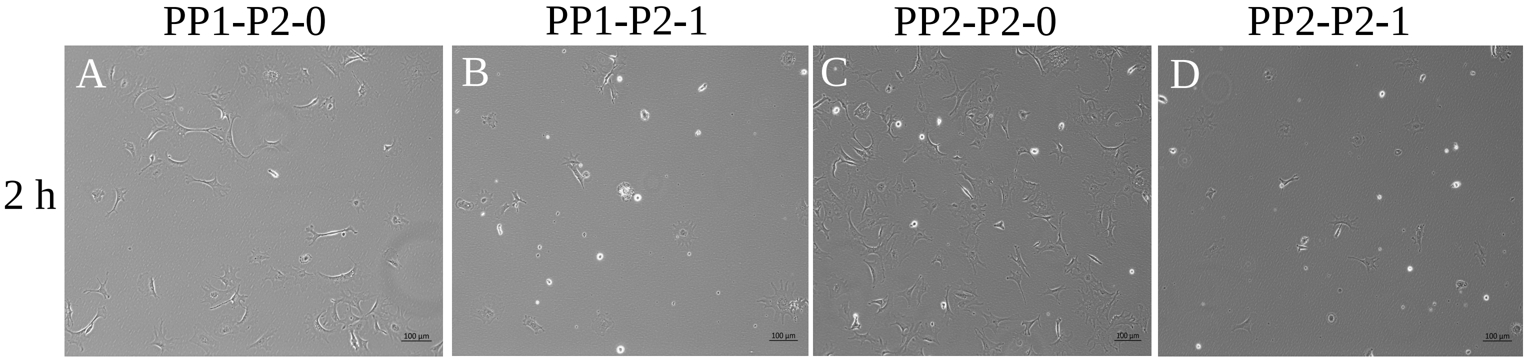

Supplementary Figure S3. Differential adhesion culture and subculture of yak SCs. PP1-P1 and PP2-P1 cells were subcultured and the cells were attached to the wall at a differential rate of 20 min. (A, C) Cells were attached to the wall. (B, D) Unadherent cells.

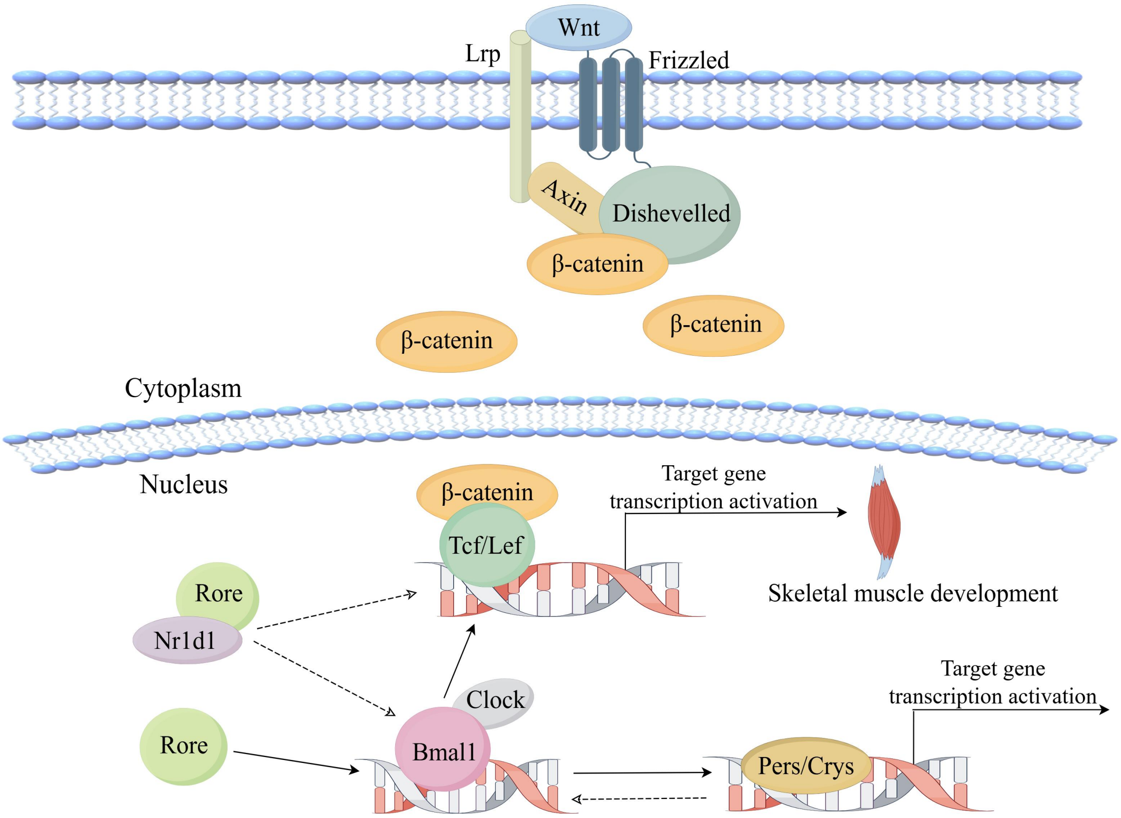

Supplementary Figure S4. Regulation of Skeletal Muscle by *NR1D1.*
Note: Solid lines indicate promotion, while dashed lines indicate inhibition (By Figdraw.)
